# Cell-Cycle Protein Expression in a Population-Based Study of Ovarian and Endometrial Cancers

**DOI:** 10.3389/fonc.2015.00025

**Published:** 2015-02-09

**Authors:** Ashley S. Felix, Mark E. Sherman, Stephen M. Hewitt, Munira Z. Gunja, Hannah P. Yang, Renata L. Cora, Vicky Boudreau, Kris Ylaya, Jolanta Lissowska, Louise A. Brinton, Nicolas Wentzensen

**Affiliations:** ^1^Hormonal and Reproductive Epidemiology Branch, Division of Cancer Epidemiology and Genetics, National Cancer Institute, National Institutes of Health, Bethesda, MD, USA; ^2^Cancer Prevention Fellowship Program, Division of Cancer Prevention, National Cancer Institute, National Institutes of Health, Bethesda, MD, USA; ^3^Breast and Gynecologic Cancer Research Group, Division of Cancer Prevention, National Cancer Institute, National Institutes of Health, Bethesda, MD, USA; ^4^Laboratory of Pathology, Center for Cancer Research, National Cancer Institute, National Institutes of Health, Bethesda, MD, USA; ^5^Tissue Array Research Program, National Cancer Institute, National Institutes of Health, Bethesda, MD, USA; ^6^Maria Sklodowska-Curie Memorial Cancer Center and Institute of Oncology, Warsaw, Poland

**Keywords:** molecular etiology, prognosis, gynecologic cancer, cell-cycle

## Abstract

Aberrant expression of cyclin-dependent kinase (CDK) inhibitors is implicated in the carcinogenesis of many cancers, including ovarian and endometrial cancers. We examined associations between CDK inhibitor expression, cancer risk factors, tumor characteristics, and survival outcomes among ovarian and endometrial cancer patients enrolled in a population-based case-control study. Expression (negative vs. positive) of three CDK inhibitors (p16, p21, and p27) and ki67 was examined with immunohistochemical staining of tissue microarrays. Logistic regression was used to estimate adjusted odds ratios (ORs) and 95% confidence intervals (CIs) for associations between biomarkers, risk factors, and tumor characteristics. Survival outcomes were only available for ovarian cancer patients and examined using Kaplan–Meier plots and Cox proportional hazards regression. Among ovarian cancer patients (*n* = 175), positive p21 expression was associated with endometrioid tumors (OR = 12.22, 95% CI = 1.45–102.78) and higher overall survival (log-rank *p* = 0.002). In Cox models adjusted for stage, grade, and histology, the association between p21 expression and overall survival was borderline significant (hazard ratio = 0.65, 95% CI = 0.42–1.05). Among endometrial cancer patients (*n* = 289), positive p21 expression was inversely associated with age (OR ≥ 65 years of age = 0.25, 95% CI = 0.07–0.84) and current smoking status (OR: 0.33, 95% CI 0.15, 0.72) compared to negative expression. Our study showed heterogeneity in expression of cell-cycle proteins associated with risk factors and tumor characteristics of gynecologic cancers. Future studies to assess these markers of etiological classification and behavior may be warranted.

## Introduction

Dysregulation of cell-cycle control mechanisms has been observed in many human cancers (1). Cell-cycle checkpoints control the timing of transitions to ensure appropriate DNA replication. Passage through the G1 checkpoint is influenced by the retinoblastoma protein (pRb), which in turn is regulated by the activity of cyclins, cyclin-dependent kinases (CDKs), and CDK inhibitors. The CDK inhibitors, p16, p21, and p27, interact with cyclin-CDK complexes to down-regulate phosphorylation of pRb, inhibiting G1 progression, and restricting cell growth. Alterations resulting in overexpression of cell-cycle stimulating proteins (cyclins or CDKs) or inactivation of inhibiting factors (CDK inhibitors or pRb) have the potential to disrupt the cell-cycle and initiate uncontrolled cell proliferation (2).

Aberrant expression of the CDK inhibitors has been frequently characterized among women with ovarian and endometrial cancers (3); however, relationships between these biomarkers with tumor characteristics and survival have offered a conflicting portrait. For example, among ovarian cancer patients, positive p16 expression has been associated with less favorable tumor characteristics by some (4–8) but not others (9–12). Furthermore, the prognostic significance of p16 is highly variable in ovarian cancer studies, with some reporting lower mortality (4, 5, 11), higher mortality (6, 8), or no association (7, 10, 12). Loss of p21 and p27 expressions is typically associated with worse grade, stage, and survival among ovarian cancer patients (13–17). Several studies have reported similarly inconsistent relationships between cell-cycle markers and tumor characteristics among women with endometrial cancer (18–27).

To our knowledge, no previous study has investigated associations between the CDK inhibitors and etiologic risk factors associated with development of these two cancers. Molecular epidemiologic studies can advance our understanding of the pathogenesis of gynecologic malignancies. Therefore, we examined associations between cell-cycle proteins, epidemiologic risk factors, tumor characteristics, and survival among ovarian and endometrial cancer patients to examine etiologic heterogeneity at the molecular level. We hypothesize that aberrant cell-cycle protein expression would be associated with risk factors for these two malignancies as well as unfavorable tumor characteristics and poor survival.

## Materials and Methods

### Study population

Data for this study are available from the population-based case-control Polish Cancer Study, which has been described previously (28, 29). Briefly, eligible cases were diagnosed between June 1, 2001 and December 30, 2003, resided in Warsaw or Lodz, Poland, and aged 20–74 at the time of diagnosis. For the current study, incident ovarian (*N* = 317) and endometrial (*N* = 551) cancer cases were ascertained through a rapid identification system coordinated by five participating Polish hospitals, which covers approximately 85% of all cases diagnosed in the two cities (28). Additionally, cancer registries in Warsaw and Lodz were used to identify cases missed by the rapid identification system. Interviewer administered questionnaires collected information on demographics, anthropometric factors, reproductive characteristics, exogenous hormone use, and cigarette smoking. Medical records were reviewed for pathology, treatment, and outcomes information. One pathologist (MES) verified the pathologic diagnosis through hematoxylin and eosin (H&E) slide review. This study was reviewed and approved by the institutional review boards (IRBS) of the U.S. National Cancer Institute (NCI), the M. Sklodowska Curie Institute of Oncology and Cancer Center in Warsaw, and the Institute of Occupational Medicine in Lodz. This study is covered by Single Project Assurances (SPAs) in Warsaw (S-009741-04) and Lodz (S-017191-01). All participants provided written informed consent for use of clinical data and archival tissue specimens.

### Pathology

Formalin-fixed paraffin-embedded tissue blocks were available for a subset of ovarian (55%) and endometrial (52%) cancer cases. Tissue microarrays (TMAs) were constructed at Yale University after H&E stained slides were retrieved to mark representative areas of each tumor. Between two and four cores, 0.6-mm in diameter were punched from donor blocks and transferred to recipient blocks. Sections were cut from each TMA block and immunohistochemical stains for p21, p27, and a dual stain for p16 and ki67 were performed at the Applied Molecular Pathology Laboratory at the NCI.

### Immunohistochemical staining and scoring

All TMA formalin-fixed paraffin-embedded sections were deparaffinized through xylene and rehydrated in graded ethanols. Antigen retrieval was carried out in a pressure cooker using DAKO citrate buffer, pH6.0 for p27, and DAKO Tris/EDTA, pH9.0 for monoclonal mouse p21 for 20 min. Endogenous peroxidase activity was quenched with 3% H202 for 10 min followed by primary antibody application of rabbit polyclonal anti-p27 (Thermo Scientific, Pittsburgh, PA, USA, clone Kip1) at 1:1000 for 30 min and mouse monoclonal anti-p21 (DAKO, Carpinteria, CA, USA, Clone SX118) at 1:25 for 2 h. Antigen–antibody complexes were detected with DAKO Env+ detection system and 3,3-diaminobenzidine, counterstained with hematoxylin, dehydrated, and coverslipped. For antibody p16/ki67, the immunohistochemical assay was performed using CINtec PLUS Kit (Westborough, MA, USA, catalog 9537) using their recommended procedures for simultaneous qualitative detection of p16 (INK4a) and Ki67 antigens modified slightly and respectively for histologic specimens. Slides were imaged on a Hamamatsu Nanozoomer and one pathologist (MES) evaluated and scored the ovarian TMA slides while two cytotechnologists evaluated the endometrial TMA slides (RLC and VB) using the SlidePath Digital Image Hub (Leica Microsystems, Dublin, Ireland). For the endometrial TMA, there was no overlap in the stains read by the two cytotechnologists – e.g., one cytotechnologist read all of the p16/ki67 stains while the second cytotechnologist read all of the p21 and p27 stains.

The percentage of stained cells (range: 0–100%) and intensity (0 = negative, 1 = weak, 2 = moderate, 3 = strong) were recorded for each marker. An overall score was calculated as the product of the percentage of stained cells and intensity, resulting in a range of 0 and 300 for each core. All p16/ki67 double stains showed strong intensity; therefore, only the percentage of stained cells was used for these markers (range: 0–100%). Protein expression was dichotomized as negative (<10) vs. positive (≥10) for all markers and representative immunohistochemical stains are shown in Figure S1 in Supplementary Material. Established IHC cut points for these markers do not exist; therefore, we chose the threshold of 10 based on the observed distribution of the overall scores in our study population and our biological hypothesis that loss of expression of the CDK inhibitors is clinically relevant. Sensitivity analyses using other cut points to denote negative and positive expression (0 vs. ≥0, 0–19 vs. ≥20) did not produce differences in associations. For each stain, we examined the correlation between multiple TMA cores using Spearman’s rank correlation statistic, which was >0.82 among ovarian cancer cases and >0.69 among endometrial cancer cases. Average and highest values of the cores were similar and the highest value of the cores was used in all analyses. Sensitivity analyses using the average of the cores were run and the results did not appreciably change.

### Statistical analysis

Correlations between markers were assessed with Spearman’s rank correlation statistic using the overall score for p21 and p27 and the percentage of stained cells for p16 and ki67 by cancer site overall and by histologic subtype among ovarian cancer patients. Relationships between p16, p21, and p27 expression, epidemiologic risk factors, and tumor characteristics were first assessed using Pearson chi-square tests. Logistic regression was used to generate multivariable adjusted odds ratios (ORs) and 95% confidence intervals (CIs) predicting positive vs. negative expression of each marker. The relationship between marker expression and epidemiologic characteristics was adjusted for age, menopausal status, menopausal hormone use, smoking status, body mass index, parity, and oral contraceptive use. The relationship between marker expression and tumor characteristics was adjusted for age, grade, stage, and histology. Information on epidemiologic risk factors was available as previously described (28). Grade and stage were available for ovarian and endometrial cancer cases. Because of small numbers, we examined grade I vs. grades II or higher, and stage I vs. stages II or higher. Ovarian histology included serous, endometrioid, clear cell, mucinous, and mixed epithelial. The predominant histology of endometrial cancers was endometrioid.

For ovarian cancer patients, we had additional information on survival from medical records. We did not examine relationships between biomarker expression and endometrial cancer outcomes given the small number of deaths. Overall survival was defined as the number of days between the date of surgery and the date of death from all causes or the date of last follow-up. Kaplan–Meier curves compared overall survival according to marker expression. When a marker was significantly associated with overall survival, we used Cox proportional hazards regression to estimate hazard ratios (HRs) and 95% CIs adjusted for stage, histology, and grade.

We generated a heat map to compare distributions of p16, p21, p27, and ki67 expression across ovarian cancer histology types and endometrioid endometrial cancer. All markers were transformed to the same scale (0–300) and the continuous value of each marker for each individual case was grouped according to histology type. The Kurman and Shih paradigm of epithelial ovarian carcinoma pathogenesis guided the categorization of ovarian cancers (30). All statistical analyses were performed using the SAS Software Package, version 9.3 (SAS Institute, Cary, NC, USA) and a two-sided *p* ≤ 0.05 was considered statistically significant.

## Results

### Study population

Table 1 shows distributions of epidemiologic and tumor characteristics among ovarian and endometrial cancer patients by TMA inclusion status. Tumor characteristics significantly differed between those included and not included on the TMA; for the most part, cases with missing grade, stage, or other histology were less likely to be included on either the ovarian or endometrial TMA. Non-smokers were more likely to be included on the endometrial TMA.

**Table 1:** **Selected characteristics of ovarian and endometrial cancer cases in the Polish Cancer Study by inclusion on the tissue microarray (TMA)**.

	Ovarian cancer			Endometrial cancer		
	Not included on TMA	Included on TMA	*p*	Not included on TMA	Included on TMA	*p*^a^
	*n* = 142	*n* = 175		*n* = 262	*n* = 289	
	*n* (%)	*n* (%)		*n* (%)	*n* (%)	
**Age**			0.47			0.22
<50	39 (27.5)	58 (33.1)		34 (13.0)	24 (8.3)	
50–54	26 (18.3)	22 (12.6)		36 (13.7)	39 (13.5)	
55–64	34 (23.9)	42 (24.0)		100 (38.2)	106 (36.7)	
65+	43 (30.3)	53 (30.3)		95 (35.1)	120 (41.5)	
**Menopausal status**			0.31			0.29
Premenopausal	33 (23.2)	50 (28.6)		43 (16.4)	36 (12.5)	
Postmenopausal	93 (65.5)	100 (57.1)		196 (74.8)	220 (76.1)	
Missing	16 (11.3)	25 (14.3)		23 (8.8)	33 (11.4)	
**Menopausal hormone use**			0.83			0.37
No	106 (74.6)	134 (76.7)		194 (74.0)	210 (72.7)	
Yes	36 (25.3)	40 (22.9)		63 (24.0)	77 (26.6)	
Missing	0 (0.0)	1 (0.6)		5 (1.9)	2 (0.7)	
**Smoking status**			0.51			0.03
Non-smoker	68 (47.9)	73 (41.7)		158 (60.3)	200 (69.2)	
Past	27 (19.0)	40 (22.9)		45 (17.2)	48 (16.6)	
Current	47 (33.1)	62 (35.4)		59 (22.5)	41 (14.2)	
**Body mass index**			0.32			0.86
<25	90 (63.4)	96 (54.9)		77 (29.4)	81 (28.0)	
25–30	34 (23.9)	50 (28.6)		93 (35.5)	113 (39.1)	
>30	16 (11.3)	28 (16.0)		89 (34.0)	92 (31.8)	
Missing	2 (1.4)	1 (0.6)		3 (1.1)	3 (1.0)	
**Parity**			0.91			0.03
Nulliparous	30 (21.1)	35 (20.0)		41 (15.6)	61 (21.1)	
1–2 Live births	103 (72.5)	127 (72.6)		188 (71.8)	208 (72.0)	
≥3 Live births	9 (6.3)	13 (7.4)		33 (12.6)	20 (6.9)	
**Oral contraceptive use**			0.74			0.10
No	128 (90.1)	162 (92.6)		243 (92.7)	277 (95.8)	
Yes	12 (8.4)	11 (6.3)		16 (6.1)	12 (4.1)	
Missing	2 (1.4)	2 (1.1)		3 (1.1)	0 (0.0)	
**Grade**			<0.0001			<0.0001
I	9 (6.3)	37 (21.1)		95 (36.3)	206 (71.3)	
≥II	44 (31.0)	129 (73.7)		37 (14.1)	80 (27.7)	
Missing	89 (62.7)	9 (5.1)		130 (49.6)	3 (1.0)	
**Stage**			<0.0001			<0.0001
I	23 (16.2)	64 (36.6)		105 (40.1)	208 (72.0)	
≥II	36 (25.3)	92 (52.6)		29 (11.1)	45 (15.6)	
Missing	83 (58.4)	19 (10.9)		128 (48.8)	36 (12.5)	
**Histology**			0.003			<0.0001
Serous	60 (42.2)	74 (42.3)		9 (3.4)	1 (0.3)	
Endometrioid	29 (20.4)	19 (10.9)		208 (79.4)	223 (77.2)	
Mucinous	10 (7.0)	10 (5.7)		3 (1.1)	1 (0.3)	
Mixed epithelial	11 (7.7)	40 (22.9)		19 (7.2)	50 (17.3)	
Clear cell	4 (2.8)	7 (4.0)		4 (1.5)	1 (0.3)	
Carcinosarcoma	–	–		5 (1.9)	10 (3.5)	
Other	28 (19.7)	25 (14.3)		14 (5.3)	3 (1.0)	

*^a^Fisher’s exact *p*-value reported when >25% of cell counts are <5*.

### Correlations between cell-cycle markers

Spearman correlations between the continuous values of all cell-cycle markers by cancer site are shown in Table 2. Among ovarian cancer patients, significant positive correlations were observed for p27 with p16 (*p* < 0.0001) and ki67 (*p* < 0.0001), but a significant inverse association between p27 and p21 was observed. Among endometrial cancer patients, we noted significant positive correlations between p27 and p16 (*p* < 0.05), p27 and p21 (*p* < 0.0001), and p27 and ki67 (*p* < 0.0001).

**Table 2:** **Spearman correlation coefficients between cell-cycle markers by cancer site**.

	p16	p21	p27	ki67
**Ovarian cancer, *n = 175***				
p16	–	−0. 11	0. 33**	0. 09
p21	–	–	−0. 45**	−0. 10
p27	–	–	–	0. 44**
ki67	–	–	–	–
**Serous ovarian, *n = 73***				
p16	–	−0. 05	0. 11	0. 03
p21	–	–	−0. 13	−0. 06
p27	–	–	–	0. 35*
ki67	–	–	–	–
**Non-serous ovarian, *n = 75 ^a^***				
p16	–	0. 09	0. 35*	0. 03
p21	–	–	−0. 25*	0. 004
p27	–	–	–	0. 44**
ki67	–	–	–	–
**Endometrial cancer, *n = 289 ^b^***				
p16	–	0. 11	0. 17*	0. 17*
p21	–	–	0. 37**	0. 41**
p27	–	–	–	0. 38**
ki67	–	–	–	–

*^a^Non-serous ovarian cancers include mixed epithelial (*n* = 39), endometrioid (*n* = 19), mucinous (*n* = 10), and clear cell (*n* = 7)*.

*^b^Endometrial tumors were predominantly endometrioid*.

*p < 0.05.

**p < 0.0001.

### Cell-cycle expression among ovarian cancer patients

Table 3 shows associations between dichotomous cell-cycle and ki67 expression, epidemiologic risk factors, and tumor characteristics among ovarian cancer cases. Positive expression of p16, p21, p27, and ki67 was observed in 79, 68, 80, and 64% of ovarian cancer patients, respectively. None of the epidemiological risk factors were significantly associated with expressions of the four markers. Compared to negative expression, positive p16 (OR = 3.15, 95% CI = 1.09–9.07) and positive ki67 (OR = 4.61, 95% CI = 1.70–12.58) were associated with higher odds of grades II and III tumors while positive p21 was associated with higher odds of endometrioid tumors (OR = 12.22, 95% CI = 1.45–102.78). Compared to negative p27 expression, positive p27 expression was inversely associated with most of the ovarian cancer histologic subtypes, including endometrioid, mucinous, and clear cell tumors.

**Table 3:** **Adjusted odds ratios (ORs) and 95% confidence intervals (CIs) of risk factors and tumor characteristics for positive vs. negative expression of cell-cycle markers among ovarian cancer patients (*n* = 175)**.

	p16			p21			p27			ki67		
	Negative (*n* = 35)	Positive (*n* = 138)	OR (95% CI)^a^	Negative (*n* = 56)	Positive (*n* = 118)	OR (95% CI)^a^	Negative (*n* = 35)	Positive (*n* = 139)	OR (95% CI)^a^	Negative (*n* = 62)	Positive (*n* = 111)	OR (95% CI)^a^
								
	*n* (%)			*n* (%)			*n* (%)			*n* (%)	
**Age**												
<50	6 (17.1)	52 (37.7)	1.00	14 (25.0)	44 (37.3)	1.00	8 (22.9)	50 (36.0)	1.00	20 (32.3)	38 (34.2)	1.00
50–54	7(20.0)	15 (10.9)	0.20 (0.04, 0.92)	5 (8.9)	17 (14.4)	2.91 (0.63, 13.47)	6 (17.1)	16 (11.5)	0.31 (0.07, 1.44)	8 (12.9)	14 (12.6)	0.69 (0.19, 2.42)
55–64	8 (22.9)	33 (23.9)	0.45 (0.09, 2.24)	16 (28.6)	25 (21.2)	1.14 (0.29, 4.37)	5 (14.3)	36 (25.9)	1.06 (0.18, 6.14)	9 (14.5)	32 (28.8)	1.43 (0.37, 5.51)
65+	14 (40.0)	38 (27.5)	0.25 (0.05, 1.29)	21 (37.5)	32 (27.1)	1.01 (0.25, 4.01)	16 (45.7)	37 (26.6)	0.36 (0.07, 1.90)	25 (40.3)	27 (24.3)	0.36 (0.09, 1.38)
*p*^b^			0.15			0.47			0.13			0.06
**Menopausal status**												
Premenopausal	6 (17.1)	44 (31.9)	1.00	7 (12.5)	43 (36.4)	1.00	8 (22.9)	42 (30.2)	1.00	19 (30.6)	31 (27.9)	1.00
Postmenopausal	22 (62.9)	76 (55.1)	1.00 (0.21, 4.77)	36 (64.3)	63 (53.4)	0.28 (0.07, 1.18)	23 (65.7)	76 (54.7)	1.07 (0.22, 5.29)	35 (56.4)	63 (56.8)	2.19 (0.61, 7.77)
*p*^b^			0.47			0.68			0.96			0.41
**Menopausal hormone use**												
No	27 (77.1)	106 (76.8)	1.00	42 (75.0)	92 (78.0)	1.00	31 (88.6)	103 (74.1)	1.00	49 (79.0)	84 (75.7)	1.00
Yes	8 (22.9)	31 (22.5)	0.68 (0.25, 1.83)	13 (23.2)	26 (22.0)	0.84 (0.35, 1.99)	4 (11.4)	35 (25.2)	2.18 (0.68, 6.98)	13 (21.0)	25 (22.5)	0.84 (0.37, 1.92)
*p*^b^			0.44			0.69			0.19			0.68
**Smoking status**												
Non-smoker	14 (40.0)	59 (42.7)	1.00	24 (42.9)	49 (41.5)	1.00	14 (40.0)	59 (42.4)	1.00	29 (46.8)	44 (39.6)	1.00
Former	7 (20.0)	31 (22.5)	1.25 (0.39, 4.01)	12 (21.4)	27 (22.9)	1.09 (0.43, 2.80)	8 (22.9)	31 (22.3)	0.86 (0.29, 2.50)	13 (21.0)	25 (22.5)	1.10 (0.44, 2.73)
Current	14 (40.0)	48 (34.8)	0.70 (0.26, 1.91)	20 (35.7)	42 (35.6)	0.82 (0.35, 1.92)	13 (37.1)	49 (35.2)	0.92 (0.34, 2.46)	20 (32.3)	42 (37.8)	1.19 (0.52, 2.69)
*p*^b^			0.59			0.83			0.96			0.92
**Body mass index**												
<25	20 (57.1)	76 (55.1)	1.00	30 (53.6)	66 (55.9)	1.00	20 (57.1)	76 (54.7)	1.00	33 (53.2)	63 (56.8)	1.00
25–30	13 (37.1)	36 (26.1)	0.75 (0.29, 1.90)	14 (25.0)	36 (30.5)	1.35 (0.57, 3.17)	12 (34.3)	38 (27.3)	1.02 (0.41, 2.53)	18 (29.0)	31 (27.9)	0.96 (0.42, 2.18)
>30	2 (5.7)	25 (18.1)	4.69 (0.90, 24.39)	11 (19.6)	16 (13.6)	0.85 (0.31, 2.35)	3 (8.6)	24 (17.3)	2.90 (0.73, 11.57)	11 (17.7)	16 (14.4)	0.68 (0.25, 1.84)
*p*^b^			0.10			0.67			0.30			0.74
**Parity**												
Nulliparous	10 (28.6)	24 (17.4)	1.00	9 (16.1)	25 (21.2)	1.00	5 (14.3)	29 (20.9)	1.00	12 (19.3)	22 (19.8)	1.00
1–2 Live births	22 (62.9)	104 (75.4)	2.31 (0.88, 6.02)	42 (75.0)	85 (72.0)	0.66 (0.26, 1.70)	27 (77.1)	100 (71.9)	0.58 (0.19, 1.74)	42 (67.7)	84 (75.7)	1.02 (0.44, 2.37)
≥3 Live births	3 (8.6)	10 (7.2)	1.50 (0.28, 8.14)	5 (8.9)	8 (6.8)	0.64 (0.13, 3.14)	3 (8.6)	10 (7.2)	0.56 (0.10, 3.32)	8 (12.9)	5 (4.5)	0.33 (0.07, 1.51)
*p*^b^			0.22			0.69			0.61			0.27
**Oral contraceptive use**												
Never	34 (97.1)	126 (91.3)	1.00	52 (92.9)	109 (92.4)	1.00	34 (97.1)	127 (91.4)	1.00	59 (95.2)	101 (91.0)	1.00
Ever	1 (2.9)	10 (7.2)	3.30 (0.35, 31.12)	2 (3.6)	9 (7.6)	2.57 (0.45, 14.72)	1 (2.9)	10 (7.2)	2.05 (0.23, 18.56)	2 (3.2)	9 (8.1)	2.93 (0.56, 15.32)
*p*^b^			0.30			0.29			0.52			0.20

	**p16**			**p21**			**p27**			**ki67**		
	**Negative (*n* = 35)**	**Positive (*n* = 138)**	**OR (95% CI)^c^**	**Negative (*n* = 56)**	**Positive (*n* = 118)**	**OR (95% CI)^c^**	**Negative (*n* = 35)**	**Positive (*n* = 139)**	**OR (95% CI)^c^**	**Negative (*n* = 62)**	**Positive (*n* = 111)**	**OR (95% CI)^c^**
								
	***n* (%)**			***n* (%)**			***n* (%)**			***n* (%)**	

**Grade**												
I	15 (42.9)	21 (15.2)	1.00	6 (10.7)	31 (26.3)	1.00	12 (34.3)	25 (18.0)	1.00	21 (33.9)	15 (13.5)	1.00
≥II	19 (54.3)	109 (79.0)	3.15 (1.09, 9.07)	50 (89.3)	78 (66.1)	0.53 (0.17, 1.67)	19 (54.3)	109 (78.4)	1.17 (0.39, 3.56)	34 (54.8)	94 (84.7)	4.61 (1.70, 12.58)
*p*^b^			0.05			0.56			0.75			0.004
**Stage**												
I	16 (45.7)	48 (34.8)	1.00	11 (19.6)	53 (44.9)	1.00	20 (57.1)	44 (31.6)	1.00	30 (48.4)	34 (30.6)	1.00
≥II	15 (42.9)	75 (54.3)	0.95 (0.36, 2.54)	36 (64.3)	55 (46.6)	0.57 (0.24, 1.39)	11 (31.4)	80 (57.5)	2.15 (0.80, 5.75)	25 (40.3)	65 (58.6)	1.52 (0.67, 3.47)
*p*^b^			0.42			0.31			0.16			0.54
**Histology**												
Serous	10 (28.6)	63 (45.6)	1.00	36 (64.3)	37 (31.4)	1.00	5 (14.3)	68 (48.9)	1.00	21 (33.9)	52 (46.8)	1.00
Endometrioid	6 (17.1)	13 (9.4)	0.56 (0.14, 2.22)	1 (1.8)	18 (15.2)	12.22 (1.45, 102.78)	7 (20.0)	12 (8.6)	0.13 (0.03, 0.60)	6 (9.7)	13 (11.7)	2.16 (0.56, 8.40)
Mucinous	5 (14.3)	5 (3.6)	0.16 (0.03, 0.96)	1 (1.8)	9 (7.6)	3.34 (0.33, 33.52)	4 (11.4)	6 (4.3)	0.13 (0.02, 0.81)	5 (8.1)	5 (4.5)	1.45 (0.29, 7.21)
Mixed epithelial	5 (14.3)	34 (24.6)	1.33 (0.38, 4.62)	13 (23.2)	27 (22.9)	1.90 (0.80, 4.48)	7 (20.0)	33 (23.7)	0.38 (0.10, 1.41)	12 (19.3)	27 (24.3)	1.09 (0.43, 2.80)
Clear cell	2 (5.7)	5 (3.6)	0.30 (0.04, 2.18)	1 (1.8)	6 (5.1)	5.91 (0.58, 60.27)	3 (8.6)	4 (2.9)	0.13 (0.02, 0.84)	4 (6.4)	3 (2.7)	0.39 (0.07, 2.16)
Other	7 (20.0)	18 (13.0)	0.38 (0.11, 1.32)	4 (7.1)	21 (17.8)	3.90 (1.13, 13.49)	9 (25.7)	16 (11.5)	0.17 (0.04, 0.64)	14 (22.6)	11 (9.9)	0.48 (0.17, 1.37)
*p*^b^			0.16			0.05			0.05			0.38

*^a^Logistic regression model predicting positive vs. negative expression of the marker adjusted for age, menopausal status, menopausal hormone use, smoking status, body mass index, parity, and oral contraceptive use*.

*^b^Chi-square *p* from logistic regression model*.

*^c^Logistic regression model predicting positive vs. negative expression of the marker adjusted for age, grade, stage, and histology*.

**Figure 1:**
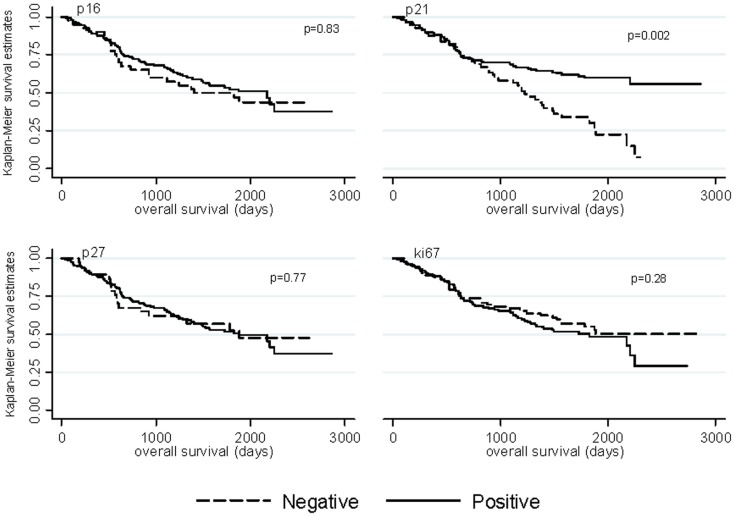
**Kaplan–Meier overall survival curves by p16, p21, p27, and ki67 expression among ovarian cancer patients**.

Median follow-up time was 4.41 years among ovarian cancer patients (range: 0.19–10.43 years) and 56% (*n* = 98) of ovarian cancer patients died. Kaplan–Meier graphs of the cell-cycle markers and overall survival are shown in Figure 1. Positive p21 expression was associated with better survival compared with negative p21 expression (log-rank *p* = 0.002). Adjustment for stage, histology, and grade attenuated the association between p21 expression and overall survival (HR = 0.66, 95% CI = 0.42–1.05). We also explored relationships between cell-cycle expression and survival among the ovarian cancer histologic subtypes. Positive p21 expression was associated with better survival among the women with endometrioid (HR = 0.02, 95% CI = 0.00–0.55) but not serous (HR = 0.97, 95% CI = 0.54–1.73) tumors in models adjusted for stage and grade. No association between p16, p27, ki67, and overall survival were observed in the overall study population (Figure 1) or in subgroups defined by stage (data not shown).

### Cell-cycle expression among endometrial cancer patients

Associations for endometrial cancer patients are described in Table 4. Positive expression of p16, p21, p27, and ki67 was observed in 89, 46, 80, and 81% of cases, respectively. Positive p21 expression decreased with older age (OR for ≥65 years: 0.28, 95% CI 0.08, 0.93) and current smoking (OR: 0.30, 95% CI 0.13, 0.68). Positive ki67 expression also decreased with older age at endometrial cancer diagnosis (OR for diagnosis 50–54 years: 0.13, 95% CI 0.02, 0.69). No significant associations between the cell-cycle or ki67 markers and endometrial tumor characteristics were observed (*p* > 0.15).

**Table 4:** **Adjusted odds ratios (ORs) and 95% confidence intervals (CIs) of risk factors and tumor characteristics for positive vs. negative expression of cell-cycle markers among endometrial cancer patients (*n* = 289)**.

	p16			p21			p27			ki67		
	Negative (*n* = 30)	Positive (*n* = 255)	OR (95% CI)^a^	Negative (*n* = 154)	Positive (*n* = 132)	OR (95% CI)^a^	Negative (*n* = 58)	Positive (*n* = 228)	OR (95% CI)^a^	Negative (*n* = 55)	Positive (*n* = 230)	OR (95% CI)^a^
								
	*n* (%)			*n* (%)			*n* (%)			*n* (%)	
**Age**												
<50	2 (6.7)	22 (8.6)	1.00	9 (5.8)	15 (11.4)	1.00	4 (6.9)	20 (8.8)	1.00	2 (3.6)	22 (9.6)	1.00
50–54	8 (26.7)	30 (11.8)	0.43 (0.07, 2.71)	23 (14.9)	16 (12.1)	0.35 (0.11, 1.12)	13 (22.4)	26 (11.4)	0.60 (0.15, 2.37)	15 (27.3)	23 (10.0)	0.13 (0.02, 0.69)
55–64	10 (33.3)	94 (36.9)	1.16 (0.16, 8.42)	50 (32.5)	54 (40.9)	0.49 (0.15, 1.59)	20 (34.5)	84 (36.8)	1.43 (0.34, 6.02)	16 (29.1)	88 (38.3)	0.53 (0.09, 3.12)
65+	10 (33.3)	109 (42.8)	1.37 (0.18, 10.67)	72 (46.8)	47 (35.6)	0.28 (0.08, 0.93)	21 (36.2)	98 (43.0)	1.76 (0.40, 7.73)	22 (40.0)	97 (42.2)	0.47 (0.08, 2.85)
*p*^b^			0.31			0.07			0.21			0.01
**Menopausal status**												
Premenopausal	5 (16.7)	31 (12.2)	1.00	18 (11.7)	18 (13.6)	1.00	7 (12.1)	29 (12.7)	1.00	8 (14.5)	28 (12.2)	1.00
Postmenopausal	23 (76.7)	194 (76.1)	0.76 (0.18, 3.14)	119 (77.3)	98 (74.2)	1.23 (0.44, 3.73)	45 (77.6)	172 (75.4)	0.55 (0.17, 1.78)	42 (76.4)	175 (76.1)	0.90 (0.28, 2.94)
*p*^b^			0.60			0.89			0.59			0.94
**Menopausal hormone use**												
No	18 (60.0)	190 (74.5)	1.00	111 (72.1)	98 (74.2)	1.00	40 (69.0)	169 (74.1)	1.00	36 (65.4)	172 (74.8)	1.00
Yes	12 (40.0)	63 (24.7)	0.54 (0.23, 1.29)	43 (27.9)	32 (24.2)	0.68 (0.37, 1.23)	17 (29.3)	58 (25.4)	1.59 (0.59, 4.32)	19 (34.6)	56 (24.3)	0.55 (0.27, 1.12)
*p*^b^			0.16			0.20			0.68			0.10
**Smoking status**												
Non-smoker	18 (60.0)	179 (70.2)	1.00	98 (63.6)	100 (75.8)	1.00	40 (69.0)	158 (69.3)	1.00	38 (69.1)	159 (69.1)	1.00
Former	7 (23.3)	41 (16.1)	0.70 (0.26, 1.90)	28 (18.2)	20 (15.2)	0.74 (0.37, 1.49)	12 (20.7)	36 (15.8)	0.89 (0.41, 1.98)	10 (18.2)	38 (16.5)	1.05 (0.45, 2.45)
Current	5 (16.7)	35 (13.7)	0.62 (0.20, 1.94)	28 (18.2)	12 (9.1)	0.30 (0.13, 0.68)	6 (10.3)	34 (14.9)	1.59 (0.59, 4.32)	7 (12.7)	33 (14.3)	0.92 (0.35, 2.45)
*p*^b^			0.62			0.01			0.60			0.98
**Body mass index**												
<25	10 (33.3)	71 (27.8)	1.00	40 (26.0)	41 (31.1)	1.00	16 (27.6)	65 (28.5)	1.00	13 (23.6)	68 (29.6)	1.00
25–30	8 (26.7)	104 (40.8)	1.75 (0.63, 4.86)	54 (35.1)	58 (43.9)	1.11 (0.60, 2.04)	20 (34.5)	92 (40.3)	0.97 (0.45, 2.09)	18 (32.7)	94 (40.9)	0.92 (0.40, 2.11)
>30	12 (40.0)	77 (30.2)	0.86 (0.32, 2.27)	58 (37.7)	32 (24.2)	0.57 (0.29, 1.11)	22 (37.9)	68 (29.8)	0.68 (0.31, 1.49)	24 (43.6)	65 (28.3)	0.48 (0.21, 1.09)
*p*^b^			0.35			0.08			0.53			0.12
**Parity**												
Nulliparous	4 (13.3)	56 (22.0)	1.00	26 (16.9)	34 (25.8)	1.00	11 (19.0)	49 (21.5)	1.00	7 (12.7)	53 (23.0)	1.00
1–2 Live births	22 (73.3)	184 (72.2)	0.51 (0.16, 1.64)	118 (76.6)	89 (67.4)	0.56 (0.30, 1.06)	41 (70.7)	166 (72.8)	0.88 (0.14, 1.79)	43 (78.2)	163 (70.9)	0.43 (0.17, 1.10)
≥3 Live births	4 (13.3)	15 (5.9)	0.18 (0.04, 0.89)	10 (6.5)	9 (6.8)	0.64 (0.21, 1.96)	6 (10.3)	13 (5.7)	0.55 (0.14, 2.24)	5 (9.1)	14 (6.1)	0.26 (0.06, 1.04)
*p*^b^			0.11			0.21			0.56			0.12
**Oral contraceptive use**												
Never	28 (93.3)	245 (96.1)	1.00	147 (95.4)	127 (96.2)	1.00	54 (93.1)	220 (96.5)	1.00	53 (96.4)	220 (95.6)	1.00
Ever	2 (6.7)	10 (3.9)	1.18 (0.20, 6.95)	7 (4.5)	5 (3.8)	1.30 (0.34, 4.87)	4 (6.9)	8 (3.5)	0.55 (0.14, 2.24)	2 (3.6)	10 (4.3)	2.79 (0.49, 15.99)
*p*^b^			0.85			0.70			0.40			0.25

	**p16**			**p21**			**p27**			**ki67**		
	**Negative (*n* = 30)**	**Positive (*n* = 255)**	**OR (95% CI)^c^**	**Negative (*n* = 154)**	**Positive (*n* = 132)**	**OR (95% CI)^c^**	**Negative (*n* = 58)**	**Positive (*n* = 228)**	**OR (95% CI)^c^**	**Negative (*n* = 55)**	**Positive (*n* = 230)**	**OR (95% CI)^c^**
								
	***n* (%)**			***n* (%)**			***n* (%)**			***n* (%)**	

**Grade**												
I	22 (73.3)	181 (71.0)	1.00	114 (74.0)	90 (68.2)	1.00	43 (74.1)	161 (70.6)	1.00	44 (72.7)	164 (71.3)	1.00
≥II	8 (26.7)	71 (27.8)	1.18 (0.48, 2.88)	37 (24.0)	42 (31.8)	1.64 (0.95, 2.83)	14 (24.1)	65 (28.5)	1.25 (0.62, 2.52)	11 (20.0)	68 (29.6)	2.06 (0.96, 4.40)
*p*^b^			0.93			0.20			0.71			0.17
**Stage**												
I	20 (66.7)	184 (72.2)	1.00	110 (71.4)	95 (72.0)	1.00	39 (67.2)	166 (72.8)	1.00	40 (72.7)	164 (71.3)	1.00
≥II	5 (16.7)	40 (15.7)	0.94 (0.32, 2.79)	23 (14.9)	22 (16.7)	1.03 (0.52, 2.06)	7 (12.1)	38 (16.7)	1.46 (0.57, 3.71)	7 (12.7)	38 (16.5)	1.29 (0.50, 3.33)
*p*^b^			0.91			0.88			0.15			0.86

*^a^Logistic regression model predicting positive vs. negative expression of the marker adjusted for age, menopausal status, menopausal hormone use, smoking status, body mass index, parity, and oral contraceptive use*.

*^b^Chi-square *p* from logistic regression model*.

*^c^Logistic regression model predicting positive vs. negative expression of the marker adjusted for age, grade, and stage*.

### Distribution of cell-cycle expressions in histologic subtypes

**Figure 2:**
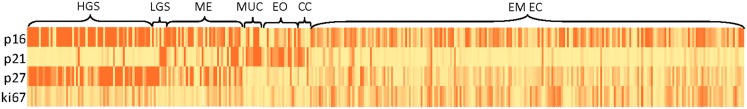
**Heat map of cell-cycle marker distributions in histologic subtypes of ovarian and endometrioid endometrial cancer patients**. Abbreviations: HGS, high-grade serous; LGS, low-grade serous; ME, mixed epithelial; MUC, mucinous; EO, endometrioid ovarian cancer; CC, clear cell; EM EC, endometrioid endometrial cancer.

The distribution of cell-cycle and ki67 expression in histologic subtypes of ovarian cancer (low and high-grade serous, mixed epithelial, mucinous, endometrioid, and clear cell) and in endometrioid endometrial cancer patients is shown in Figure 2. Cells of light yellow indicate negative or low expression while dark orange cells indicate positive or high expression of the biomarker. Women with non-serous ovarian histology (mucinous, endometrioid, and clear cell tumors) had similar cell-cycle protein expression patterns: expression of p16, p27, and ki67 were negative while p21 expression was positive among these three subgroups. Serous ovarian cancers had a unique expression pattern dependent on grade: high-grade serous tumors were characterized by negative p21 expression while some low-grade serous tumors showed positive p21 expression. The mixed epithelial tumors had expression patterns intermediate to the serous and other non-serous subtypes, with positive p16 expression and negative expression of the other markers. Cell-cycle expression patterns among the endometrioid endometrial cancers showed intermediate expression of p16 and relatively low expression of the other markers.

## Discussion

In this population-based study, we examined relationships between CDK inhibitors, epidemiologic risk factors, and tumor characteristics among ovarian and endometrial cancer patients. To our knowledge, this is the first study exploring relationships between these biomarkers and etiologic factors related to these gynecologic malignancies. The recently completed Cancer Genome Atlas (TCGA) studies of ovarian and endometrial cancers (31, 32) have provided ample evidence for molecular heterogeneity within histologic subtypes of these malignancies (31, 32); therefore, assessing relationships between molecular biomarkers and epidemiologic factors may reveal etiologic pathways beyond that of risk factor associations with histologic subtypes.

A large body of literature supports the notion that dysregulation of cell-cycle control, particularly the transition from G1 to S phase, is an important prerequisite for development of many epithelial malignancies (33). This transition requires phosphorylation of the pRb, which in turn is controlled by the activity of several classes of proteins, including cyclins, CDKs, and CDK inhibitors. This latter group of proteins, which include p16, p21, and p27, acts as negative regulators of the cell-cycle by preventing phosphorylation of pRb and arresting progression of the cell-cycle. The TCGA analysis of high-grade serous ovarian cancers reported that the Rb pathway was deregulated in 67% of cases (31). Moreover, a recently engineered mouse model recapitulating initiation and progression of serous epithelial ovarian cancers showed that alterations in the Rb pathway were sufficient to induce these tumors (34). Taken together, these findings highlight the overall importance of this pathway in ovarian carcinogenesis. Although the TCGA analysis of endometrial cancer did not identify the Rb pathway as a commonly altered target (32), endometrial cancer case-series have shown frequent alterations in key players of the Rb pathway (18–27).

In our study, most of the established risk factors for ovarian and endometrial cancers were not related to expression of the CDK inhibitors. Similar to previous ovarian cancer studies (13–15, 35, 36), we observed that positive p21 expression was associated with well-differentiated, early stage, non-serous ovarian cancer subtypes, and better survival whereas positive p16 expression was associated with poorly differentiated tumors (3, 6, 12, 13). Consistent with some previous studies (10–12), and in contrast to others (4, 5, 8), we did not detect associations between p16 expression with either stage or histology. Furthermore, no association between p16 and ovarian cancer survival was observed in our study; however, previous studies have shown that positive p16 expression is related to both lower (4, 5, 11) and higher mortality (6, 8). The contradictory findings related to p16 may be due, in part, to differences in staining protocols, cut-off values for p16 expression, and characteristics of the study populations examined. Several mechanisms have been described for overexpression of p16: p16 is a marker of aging and cellular stress, which signals pRb to halt the cell-cycle and proliferation given negative feedback interactions. In this scenario, overexpression of p16 would be expected among lower grade and early stage tumors. Conversely, overexpression of p16 could indicate downstream disruption of pRb signaling – if pRb has been inactivated by other mechanisms, negative regulation of p16 will not occur, resulting in an accumulation of p16. In this case, overexpression of p16 might be an indicator of more aggressive tumor characteristics. Furthermore, recent data suggest that p16 has dual biological roles as tumor suppressor gene and oncogene, which may in part account for the inconsistent findings (37).

p27 expression has variably been associated with better tumor characteristics and improved survival in some ovarian case-series (17, 38) while others have observed poor tumor characteristics and unfavorable survival (14, 39, 40). Our results agree with the latter group of studies, as we observed that p27 expression tended to be positive in advanced stage and serous ovarian tumors. p27 likely has a complex function in regulating the cell-cycle, which may explain the inconsistent observations.

In our series of mostly endometrioid endometrial carcinomas, we did not observe significant associations between cell-cycle expression and endometrial tumor characteristics, which agrees with some (22, 23, 41) but not other studies (19, 21, 26, 27, 42–44). Some have reported that overexpression of p16, p21, or p27 was significantly associated with poorly differentiated tumors (26, 43, 44), advanced stage (43), and worse survival (19, 42) among endometrial cancer patients. Conversely, Salvesen and colleagues (27) showed that loss of nuclear p16 was associated with poor tumor characteristics including, advanced stage, serous or clear cell histology, poorly differentiated tumors, and worse survival.

In addition, we compared expression across the histologic subtypes using a heat map to visually assess staining patterns. Non-serous ovarian tumors, including clear cell, endometrioid, and mixed tumors, displayed similar patterns of expression of p16, p21, p27, and ki67 while serous ovarian tumors showed opposite expression patterns of the CDK inhibitors. Endometrioid endometrial cancers, which made up of 99% of endometrial cancer cases included on the TMA, had an expression profile intermediate to that of non-serous and serous ovarian cancers, with high expression of p16 and low expression of the other markers. These patterns likely underscore differences in the pathobiology of these tumors and potentially have therapeutic implications (45).

Our study had several strengths including a relatively large number of cases for each tumor site with tumor tissue, availability of epidemiologic risk factor data, and high quality pathology data, as well as uniform evaluation of the immunostains. Some limitations of our analysis, which can be addressed in future studies, include limited numbers of cases within histologic subgroups and limited diversity of endometrial cancer subtypes. Furthermore, studies that examine ratios between stimulatory and inhibitory proteins of the cell-cycle e.g., cyclins and CDK inhibitors, may provide additional information beyond expression of individual proteins (46).

In conclusion, this is the first study to evaluate associations between cell-cycle biomarkers, risk factors, and tumor characteristics for two gynecologic malignancies. Our study suggests that the CDK inhibitors p16, p21, and p27 are minimally associated with epidemiologic risk factors for development of these tumors. Significant associations between CDK inhibitors, tumor characteristics, and survival were observed and support the growing body of literature related to the CDK/RB pathway for prognosis in gynecological cancers.

## Conflict of Interest Statement

The authors declare that the research was conducted in the absence of any commercial or financial relationships that could be construed as a potential conflict of interest.

## Supplementary Material

The Supplementary Material for this article can be found online at http://www.frontiersin.org/Journal/10.3389/fonc.2015.00025/abstract
