# Supplementary figures and images for: Cell-Cycle Protein Expression in a Population-Based Study of Ovarian and Endometrial Cancers
Source: Front Oncol. 2015 Feb 9;5:25. doi: 10.3389/fonc.2015.00025 (PMC4321403; doi:10.3389/fonc.2015.00025)

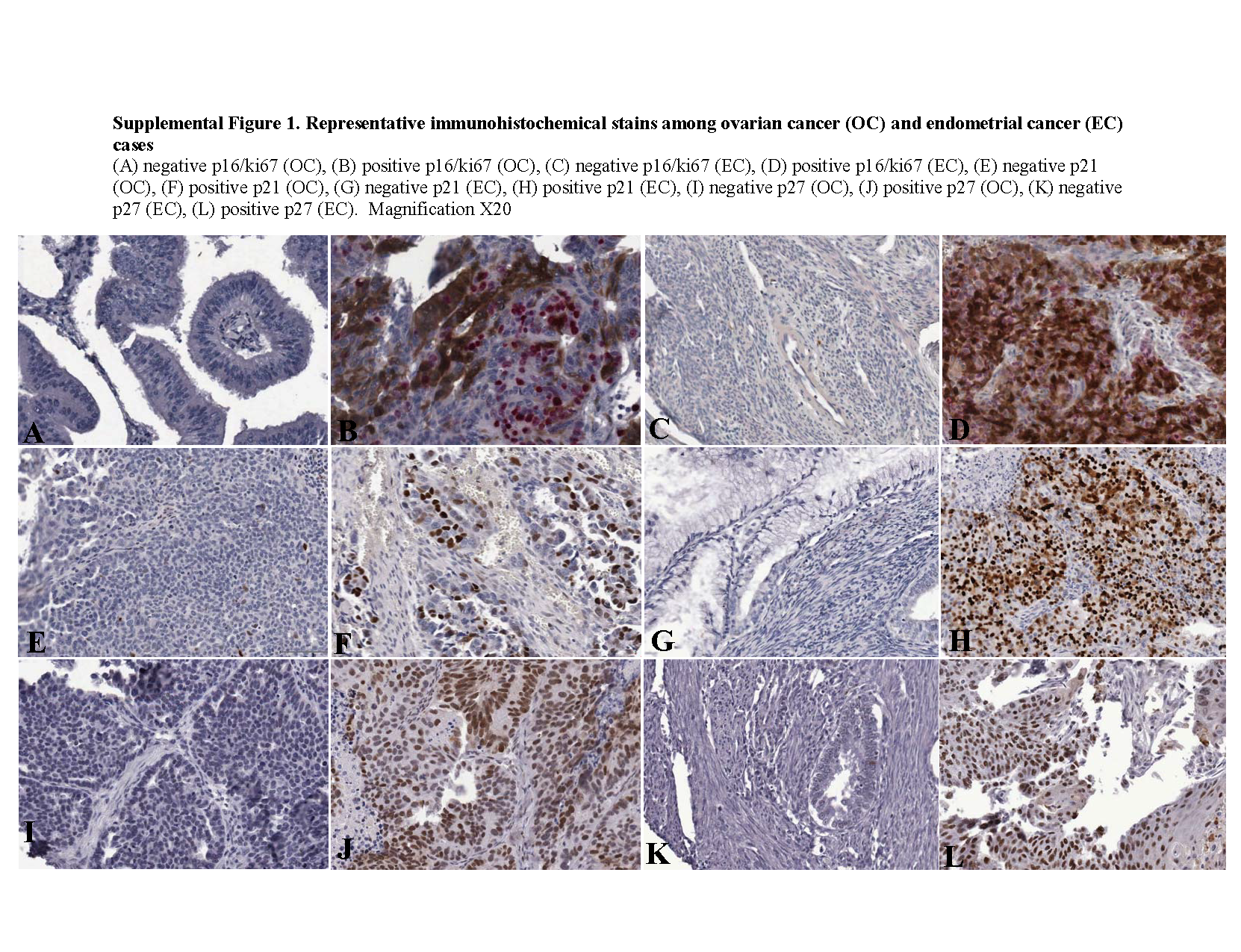

Supplement: Supplementary file 1 [file Image_1.TIF]
